# Blending an e-learning package into a problem-based learning module

**DOI:** 10.5116/ijme.5787.617d

**Published:** 2016-08-28

**Authors:** Gareth Thomas, Venugopal Duddu, Richard Gater

**Affiliations:** 1 Lancashire Care NHS Foundation Trust, Lancashire, UK; 2 Institute of Medical Psychology, Asha Hospital, Hyderabad, India; 3 Faculty of Medical and Human Sciences, University of Manchester, UK

**Keywords:** E- learning, problem-based learning, module, UK Medical School

## Introduction

Many medical schools have adopted a PBL (Problem Based Learning) approach to deliver their Undergraduate curricula. In doing so, many have adapted the PBL model in innovative ways. The aim of this article is to share the experiences of blending e-learning within an undergraduate Problem Based Learning (PBL) module at Manchester Medical School, UK.

PBL is a student centred approach that facilitates learning through the experience of problem solving. The overall goals of PBL are to help students develop flexible knowledge, effective problem solving skills, self-directed learning, effective collaboration skills and intrinsic motivation.^1^ Most PBL programs organise students into facilitated groups that meet at regular (usually weekly) intervals to refresh existing knowledge, identify new learning objectives, work towards achieving them and share their learning with peer group members. The role of the facilitator is to support the learning process whilst encouraging them to extend their understanding.^1^

PBL can be used to deliver the entire curriculum as well as individual courses/modules. Increasingly, medical schools have developed some variations in the PBL model. One specific variation is through the use of e-learning. Blending the e-learning with PBL has been shown to make a positive contribution to student satisfaction and achievement^2^^,^^3^ and provides teaching aids for difficult study topics.^4^

### PBL at Manchester Medical School (UK)

In 1994 the University of Manchester medical school introduced PBL within its medical curriculum. Feedback regarding PBL was initially positive, but as time progressed, there was reported low student satisfaction in many aspects of the program. It was felt that this was partly related to the lack of clear guidance, direction and support during the PBL process. The School responded by undertaking a pilot revamp of the year 4 ‘Mind and Movement’ module. This module lasts a total of 12 weeks, with four weeks in Psychiatry, Neurology and Orthopaedics/Rheumatology with students completing weekly PBL cases.

The revamp included the introduction of training for the PBL facilitators to be more proactive during PBL sessions and some experts reviewed the PBL booklets to include focused cases with more focussed and clear Intended Learning Objectives (ILOs). A major change was the introduction of a battery of e-learning resources to complement the PBL. Each student was given an iPad to access the new e-learning material. The e-learning materials consisted of three sets of online lectures which were developed for each weekly PBL topic and included brief introductory and summary lectures (15-30 minutes) delivered by a local Consultant, and a single in-depth lecture delivered by a renowned academic expert (45-60 minutes). In addition, supplementary clinical cases were developed for students to read and had videos to watch with a view to enhance the exposure to different clinical presentations. The e-learning also had links to resources such as clinical guidelines. Finally, some formative multiple-choice questions were available. Once the student completed the question, immediate feedback was presented.

### Students’ views and experiences

Students were generally satisfied with the quality and availability of e-resources to augment their PBL learning, but there was variability in how students used the e-resources - for example, some preferred to listen to the online introductory and expert lectures at the beginning of the PBL week, while others found formative assessments more useful. Some appreciated the ability to refine their conceptual learning by comparing the index PBL case with online supplementary cases. Consistent with expectations and the literature, e-learning provided a more student-centric approach^5^ and gave the opportunity to provide teaching aids for particularly difficult study topics.^3^ These positives were aided by the ease of use, access, consistency & transferability (uniformity of teaching across sites), and the School's validation of the e-resources provided. 

The students highlighted some important logistical and content considerations when blending e-learning within PBL. It was recommended that the e-resources should be uniformly available to students across different teaching sites, and accessible through a single teaching platform. Additionally, the students reported that Wi-Fi should be available in their teaching areas to enable access to the resources. This is also true for the home environment, as some students did not have access to Wi-Fi, which would disadvantage students.

The students highlighted that although they were taught over a wide geographical area, having access to common video lectures from experts in a particular field instilled confidence in the material. The students reported however that the inability to personally interact with the teachers was restrictive to their learning. Additionally, students preferred that the lectures had a practical clinical focus, which followed the patient’s journey throughout their care.

The supplementary e-cases were anticipated to help students to refine their concepts by comparing these with the main PBL case. Only a few students used this resource as intended as they were seen as additional rather than an essential resource. It was felt that there is a need to reconsider the terminology used to describe this set of resources, to encourage students to study these too. Additionally, integration of these e-cases with the clinical placements and how they relate to the weekly PBL topic would be useful to enhance student learning.

The formative MCQs were well received, particularly the immediate feedback to identify weaknesses. Students specifically highlighted that MCQs helped to prepare for examinations if they were in the style of the examination.

Finally, the need for a comprehensive induction program on how to use the e-resources was highlighted to enable the students to get the best use of the e-learning.

## Conclusions

The development of e-resources to augment the PBL module is a positive development by Manchester Medical School, although there are challenges as outlined. The findings are in line with similar adaptations of PBL programs,^3^ and supports increased use of e-learning tools in an Undergraduate medical curriculum. Although the students were satisfied with this pilot, it remains to be seen whether this translates into improvements with the overall satisfaction with the Undergraduate course itself.

### Conflicts of Interest

The authors declare that they have no conflict of interest.
